# Bettenkapazitätssteuerung in Zeiten der COVID-19-Pandemie: Eine simulationsbasierte Prognose der Normal- und Intensivstationsbetten anhand der deskriptiven Daten des Universitätsklinikums Augsburg

**DOI:** 10.1007/s00101-020-00830-6

**Published:** 2020-08-21

**Authors:** C. Römmele, T. Neidel, J. Heins, S. Heider, V. Otten, A. Ebigbo, T. Weber, M. Müller, O. Spring, G. Braun, M. Wittmann, J. Schoenfelder, A. R. Heller, H. Messmann, J. O. Brunner

**Affiliations:** 1 Universitätsklinikum Augsburg, Stenglinstraße 2, 86156 Augsburg, Deutschland; 2 Universitäres Zentrum für Gesundheitswissenschaften am Klinikum Augsburg (UNIKA-T), Wirtschaftswissenschaftliche Fakultät, Universität Augsburg, Neusässer Straße 47, 86159 Augsburg, Deutschland; 3 Führungsgruppe Katastrophenschutz, Zweckverband Rettungsdienst und Feuerwehralarmierung Augsburg, 86143 Augsburg, Deutschland

**Keywords:** Prognose, SARS-CoV‑2, Simulationsmodell, Monte-Carlo-Simulation, Krankenhauskapazität, Forecast, Monte Carlo simulation, Forecasting tool, Severe acute respiratory syndrome coronavirus 2, Hospital bed capacity

## Abstract

**Hintergrund:**

Die COVID-19-Pandemie zeichnet sich durch einen sich langsam aufbauenden Bedarf von Ressourcen des Gesundheitswesens mit lokalen Hotspots aus und erzeugt dadurch enorme Probleme. Für die Krankenhäuser liegt eine der größten Herausforderungen in der Vorhaltung von Bettenkapazitäten, insbesondere da der Bedarf schwer vorherzusehen ist.

**Fragestellung:**

Um den Entscheidungsträgern eine Hilfestellung zu geben, wurden mehrere simulationsbasierte Prognosen für die benötigte Bettenkapazität am Universitätsklinikum Augsburg durchgeführt, um bei variablen Pandemieverläufen die benötigten Bettenkapazitäten abschätzen zu können.

**Methode:**

Als Input dienen aktuelle Erkenntnisse über den Verlauf der Ausbreitung, insbesondere die Wachstumsrate an kumulierten Neuinfektionen pro Tag. Zur Abbildung von Unsicherheit werden mittels Verteilungsfunktionen, basierend auf Realdaten der Wachstumsrate, die Verweildauer sowie der Anteil der hospitalisierten COVID-19-Patienten im Einzugsgebiet modelliert. Im Anschluss erfolgt eine Monte-Carlo-Simulation, die eine Abschätzung der benötigten Bettenkapazitäten für mehrere Tage in der Zukunft erlaubt.

**Ergebnisse:**

Mithilfe von mehreren simulationsbasierten Kapazitätsprognosen im Zeitraum vom 28.03.2020 bis zum 08.06.2020 konnte die benötigte Intensiv- und Normalbettenkapazität am Universitätsklinikum Augsburg sowie im Rettungsdienstbereich Augsburg mit einer hohen Zuverlässigkeit prognostiziert werden.

**Schlussfolgerung:**

Mithilfe des entwickelten Simulationsmodells zur Abschätzung der benötigten Bettenkapazität kann den Kliniken und dem Katastrophenschutz eine Hilfestellung zur Abschätzung der kurzfristigen Entwicklung des Kapazitätsbedarfs für Verdachtsfälle sowie bestätigte COVID-19-Patienten gegeben werden. Der operative Einsatz der Methode am Universitätsklinikum Augsburg zeigte verlässliche Ergebnisse.

## Abstract (en)

**Background:**

Following the regional outbreak in China, severe acute respiratory syndrome coronavirus 2 (SARS-CoV-2) has spread all over the world, presenting the healthcare systems with huge challenges worldwide. In Germany the coronavirus diseases 2019 (COVID-19) pandemic has resulted in a slowly growing demand for health care with a sudden occurrence of regional hotspots. This leads to an unpredictable situation for many hospitals, leaving the question of how many bed resources are needed to cope with the surge of COVID-19 patients.

**Objective:**

In this study we created a simulation-based prognostic tool that provides the management of the University Hospital of Augsburg and the civil protection services with the necessary information to plan and guide the disaster response to the ongoing pandemic. Especially the number of beds needed on isolation wards and intensive care units (ICU) are the biggest concerns. The focus should lie not only on the confirmed cases as the patients with suspected COVID-19 are in need of the same resources.

**Material and methods:**

For the input we used the latest information provided by governmental institutions about the spreading of the disease, with a special focus on the growth rate of the cumulative number of cases. Due to the dynamics of the current situation, these data can be highly variable. To minimize the influence of this variance, we designed distribution functions for the parameters growth rate, length of stay in hospital and the proportion of infected people who need to be hospitalized in our area of responsibility. Using this input, we started a Monte Carlo simulation with 10,000 runs to predict the range of the number of hospital beds needed within the coming days and compared it with the available resources.

**Results:**

Since 2 February 2020 a total of 306 patients were treated with suspected or confirmed COVID-19 at this university hospital. Of these 84 needed treatment on the ICU. With the help of several simulation-based forecasts, the required ICU and normal bed capacity at Augsburg University Hospital and the Augsburg ambulance service in the period from 28 March 2020 to 8 June 2020 could be predicted with a high degree of reliability. Simulations that were run before the impact of the restrictions in daily life showed that we would have run out of ICU bed capacity within approximately 1 month.

**Conclusion:**

Our simulation-based prognosis of the health care capacities needed helps the management of the hospital and the civil protection service to make reasonable decisions and adapt the disaster response to the realistic needs. At the same time the forecasts create the possibility to plan the strategic response days and weeks in advance. The tool presented in this study is, as far as we know, the only one accounting not only for confirmed COVID-19 cases but also for suspected COVID-19 patients. Additionally, the few input parameters used are easy to access and can be easily adapted to other healthcare systems.

## Hinführung

Aus dem initial lokalen Ausbruch der „coronavirus disease 2019“ (COVID-19) entwickelte sich eine Pandemie mit über 2 Millionen Erkrankten weltweit (Stand 16.04.2020) [5]. In Deutschland existiert eine sehr heterogene Lage mit Regionen ohne größere Auswirkungen und lokalen Hotspots, in denen die Krankenhauskapazitäten an ihre Belastungsgrenzen stoßen. Für die Kliniken und den Katastrophenschutz sind Abschätzungen zur Lageentwicklung essenziell, um durch Steigerungen der Kapazitäten die Patientenversorgung zu gewährleisten. Gleichzeitig muss eine Verschwendung von Ressourcen verhindert werden.

## Einleitung

### Kapazitätsplanung in Zeiten der Pandemie

Die klinische Bandbreite von COVID-19 kann von einem asymptomatischen Verlauf bis zu einem Lungenversagen („acute respiratory distress syndrome“, ARDS) durch die virale Pneumonie mit Beatmungspflichtigkeit oder gar Exitus letalis reichen [15]. Die Übertragung erfolgt überwiegend über eine Tröpfcheninfektion [14]. Durch die bestehende Infektiosität noch vor dem Ausbilden von Symptomen konnte sich das Virus erfolgreich rasch verbreiten und zu einer Pandemie führen [11]. Hierdurch kam es in mehreren Regionen der Welt zu dramatischen Situationen und letztlich einem Kollaps des Gesundheitssystems vor Ort. Dieser hatte zur Folge, dass nicht jedem Patienten die eigentlich notwendige medizinische Behandlung aus Kapazitätsgründen zuteilwerden konnte. Auch dies trägt zu der regional teilweisen hohen Mortalität der COVID-19-Erkrankung bei. In Vorbereitung der Pandemie wurde in Deutschland frühzeitig begonnen, das Gesundheitswesen auf die mögliche Belastung vorzubereiten. Die Krankenhäuser müssen hierbei insbesondere ihre Normalstationsbetten mit Isolationsmöglichkeit und die Kapazitäten ihrer Intensivstationen mit Beatmungsmöglichkeit steigern. Ein Fallstrick der Krankenhäuser liegt jedoch in der Abschätzung der kurzfristigen Kapazitätsbelastung durch Patienten mit COVID-19, da hierdurch enorme finanzielle und personelle Kapazitäten gebunden werden. Hier müssen aufgrund des lokalen Krankheitsgeschehens regional große Zahlenunterschiede berücksichtigt werden. Weiterhin muss bedacht werden, dass, solange die Anzahl der Infizierten in dem Einzugsgebiet eines Krankenhauses ein gewisses Maß nicht überschreitet, zahlreiche Kapazitäten durch klinische Verdachtsfälle gebunden werden, trotz letztlich geringer tatsächlicher COVID-19-Erkrankungen.

Um der lokalen Corona Task Force am Universitätsklinikum Augsburg bei der Planung und Vorhersage der zu erwartenden Kapazitätsbelastung durch COVID-19-Patienten zu helfen, wurden daher mehrere simulationsbasierte Prognosen für die kurzfristig benötigte Bettenkapazität unter Unsicherheit durchgeführt. Mit diesen sollen die Anzahl der Krankenhausbetten auf Normal- sowie Intensivstation für verschiedene Pandemieverläufe vorhergesagt werden.

## Methoden

Das Ziel des Modells ist, die zukünftige Bettenbelegung auf der COVID-Normalstation sowie der COVID-Intensivstation zu bestimmen. Hierfür wurde ein stochastisches Simulationsmodell entwickelt, das mit relativ wenigen Input-Parametern die voraussichtliche Bettenbelegung der nächsten Tage, inklusive der jeweiligen Eintrittswahrscheinlichkeiten, in Form einer Verteilungsfunktion berechnet. Das Modell beinhaltet eine sog. Monte-Carlo-Simulation [8].

### Monte-Carlo-Simulation

Für das Verfahren werden stochastische Verteilungen für die benötigten Eingabeparameter angenommen. Die Parameter beruhen auf aktuellen Erkenntnissen zur Ausbreitung der Pandemie sowie auf Realdaten der Versorgungssituation am Universitätsklinikum Augsburg. Die Parameter lauten: die *Wachstumsrate an kumulierten Neuinfektionen pro Tag*, der *Anteil der Neuinfizierten*, die eine Behandlung auf der Normal- oder Intensivstation benötigen, sowie die *Verweildauer* auf einer Station. Für jeden dieser Parameter wurde eine Dreiecksverteilung gebildet. Eine Dreiecksverteilung ist eine stetige Wahrscheinlichkeitsverteilung, bei der die 3 Parameter Minimalwert, Maximalwert und der wahrscheinlichste Wert (Modus), übergeben werden. Die Wachstumsrate wurde in den ersten Versionen des Modells durch ein eigens entwickeltes Konstrukt abgebildet und später durch den vom Robert Koch-Institut verwendeten Replikationsfaktor $R_{t}$ [10] ersetzt. Nach der Errechnung des aktuellen Replikationsfaktors im Einzugsgebiet ist es möglich, die Anzahl der Neuinfizierten für ein (z. B. ein gleichbleibendes Infektionsgeschehen) oder mehrere mögliche zukünftige Szenarien zu errechnen. Nachdem die Eingabeparameter bestimmt wurden, werden im Modell für jeden Simulationsdurchlauf Zufallszahlen für die jeweiligen Verteilungen gezogen. Für die einzelnen Durchläufe lassen sich anhand eines gegebenen Infektionsgeschehens die Anzahl der Neuinfizierten im Einzugsgebiet und die daraus voraussichtlich entstehenden Neuaufnahmen auf der Intensiv- und Normalstation berechnen. Jeder Neuaufnahme wird aus der Verweildauerverteilung eine Bleibedauer auf der jeweiligen Station zugewiesen. Um Aufnahmen aus der Vergangenheit und deren Verweildauer in der Zukunft zu berücksichtigen, ist eine Aufwärmphase für den unmittelbaren Zeitraum vor dem Stichtag der Simulation notwendig. Aus der Verweildauer und den Neuaufnahmen lässt sich letztendlich die erwartete benötigte Bettenkapazität berechnen. Dieser Prozess wird 10.000-mal wiederholt, und in jedem Simulationsdurchlauf werden neue Zufallszahlen aus den Verteilungen gezogen. Das Modell wurde in Microsoft Excel unter Verwendung des Add-ins ModelRisk von Vose Software *mit einer eigens in VBA programmierten Schnittstelle zur Datenaufbereitung *implementiert. Die Input-Parameter sind für den Anwender flexibel einstellbar, welche dem Anwender verschiedenen Szenarien mit einem fallenden, stagnierenden oder steigenden Trend der Bettenbelegung vorhersagen. Anschließend erhält der Anwender eine Verteilungsfunktion für die zuvor definierten Ausgabeparameter *benötigte Bettenkapazität* pro zukünftigem Prognosetag und die *maximal benötige Bettenkapazität* innerhalb des Prognosehorizonts. Stochastische Auswertungen wie die Bestimmung der Mittelwerte oder der Quantile der verschiedenen Verteilungen der Ausgabeparameter können einfach ermittelt werden. Fortlaufend können (müssen) die Verteilungsfunktionen der Eingabeparameter anhand aktueller Erkenntnisse angepasst werden, da beispielsweise die Verweildauer oder auch die Wachstumsrate an kumulierten Neuinfektionen der Pandemie (insbesondere durch eingeführte Maßnahmen) sich im Laufe der Zeit verändern können. Damit kann das Modell weiter adjustiert werden.

### Vergleich mit realen Falldaten

Die getätigten Prognosen wurden dann sekundär mit den tatsächlichen am Universitätsklinikum Augsburg erhobenen deskriptiven Daten verglichen. Hierfür wurden ab der Vorstellung der ersten COVID-19-Verdachtsfälle am 02.02.2020 bis einschließlich zum 12.04.2020 retrospektiv alle COVID-19-Verdachtsfälle sowie positiven COVID-19-Patienten erfasst.

Eine positive Begutachtung des Forschungsvorhabens 2020-13 erfolgte durch die lokale Beratungskommission für klinische Forschung des Universitätsklinikums Augsburg.

## Ergebnisse

### COVID-Patienten am Universitätsklinikum Augsburg

Am Universitätsklinikum wurden vom 02.02.2020 bis einschließlich 12.04.2020 insgesamt 306 Patienten mit dem Verdacht oder einer bestätigten COVID-19-Erkrankung auf einer Normalstation oder einer Intensivstation aufgenommen. Das mediane Alter der Patienten lag bei 69 Jahren, 40 % hiervon waren weiblich. Eine Infektion mit SARS-CoV‑2 bestätigte sich letztlich bei 100 (33 %) Patienten. 247 Patienten wurden mit Verdacht auf COVID-19 auf eine COVID-Normalstation aufgenommen. Hierbei wurde bei 77 Patienten (31 %) eine COVID-19-Erkrankung nachgewiesen. Die mediane Verweildauer auf einer COVID-Normalstation lag bei 4 Tagen für COVID-19 nachgewiesene und bei 2 Tagen für ausgeschlossene COVID-19 Patienten. 84 der 306 (27 %) Patienten benötigten im Verlauf ihres Aufenthalts eine intensivmedizinische Betreuung. Von diesen 84 konnte bei 38 Patienten (45 %) eine COVID-19-Erkrankung nachgewiesen werden. 50 % der an COVID-19 erkrankten Intensivpatienten benötigten eine invasive Beatmung. Insgesamt 8 von 100 (8 %) Patienten mit nachgewiesener COVID-19 Erkrankung verstarben während des stationären Aufenthalts, hiervon 1 Patient (1 %) auf COVID-Normalstation, welcher eine intensivmedizinische Behandlung ablehnte, und 7 (18 %) auf COVID-Intensivstation (Tab. 1).

**Table 1:** 

	Patienten Gesamt	COVID-19	Nicht-COVID-19
*COVID-Normal* *+* *Intensivstationen n [%]*	*306 [100* *%]*	*100 [33* *%]*	*206 [67* *%]*
Alter [in Jahren], Median [IQR]	69 [55–79]	65 [52–76]	72 [56–81]
Anteil, weiblich, *n* [%]	122 [40 %]	33 [33 %]	89 [43 %]
Verweildauer von bisher entlassenen Pat. [d] Median [IQR]	2 [1–3]	5 [3–7]	2 [1–2]
*COVID-Normalstation [%]*	*247 [81* *%]*	*77 [31* *%]*	*170 [69* *%]*
Alter, Median [IQR]	70 [54–80]	65 [51–76]	73 [56–82]
Anteil, weiblich *n* [%]	101 [41 %]	29 [38 %]	72 [42 %]
Verlegung auf Intensivstation *n* [%]	18 [7 %]	12 [16 %]	6 [4 %]
Exitus letalis, *n* [%]	1 [0 %]	1 [1 %]	0 [0 %]
Entlassung, *n* [%]	198 [80 %]	44 [57 %]	154 [91 %]
Summe Verlegungen u. Entlassungen	217 [88 %]	57 [74 %]	160 [94 %]
Verweildauer von verlegten u. entlassenen Pat. [d] Median [IQR]	2 [1–3]	4 [2–7]	2 [1–2]
*COVID-Intensivstation n [%]*	*84 [27* *%]*	*38 [45* *%]*	*46 [55* *%]*
Alter [in Jahren], Median [IQR]	68 [58–77]	67 [60–74]	70 [58–78]
Anteil, weiblich, *n* [%]	32 [38 %]	12 [32 %]	20 [43 %]
NIV/High flow, *n* [%]	26 [31 %]	10 [26 %]	16 [35 %]
Invasive Beatmung, *n* [%]	27 [32 %]	19 [50 %]	8 [17 %]
Verlegung auf Normalstation, *n* [%]	31 [37 %]	12 [32 %]	19 [41 %]
Exitus letalis *n* [%]	7 [8 %]	7 [18 %]	0 [0 %]
Entlassung nach Hause, *n* [%]	3 [4 %]	3 [8 %]	0 [0 %]
Summe, Verlegungen u. Entlassungen	62 [74 %]	22 [58 %]	40 [87 %]

Die Anzahl der Patienten auf COVID-Normalstation sowie -Intensivstation ergibt in der Summe >100 %, da Patienten auf beiden Stationen behandelt wurden. Aufgrund der noch sehr geringen Fallzahl an Patienten, die von der Intensivstation entlassen wurden, wurde die Verweildauer für diese Patienten nicht erhoben

*n* Anzahl, *IQR* Interquartilsabstand, *d* Tage

### Input-Parameter von Verdachts- und bestätigten Fällen

Basierend auf den erhobenen Daten wurden die Verteilungen bestimmt, die für die Parameter des Modells notwendig sind. Einer der wichtigsten Parameter ist hierbei die Entwicklung der kumulierten Neuinfektionen pro Tag. Diese wird auf Basis der gemeldeten COVID-19-Fälle je Landkreis vom Bayerischen Landesamt für Gesundheit und Lebensmittelsicherheit [1] für das Einzugsgebiet des Universitätsklinikums berechnet. Das Einzugsgebiet wird durch das jeweilig anteilige Patientenaufkommen aus dem eigenen und dem der angrenzenden Landkreise bestimmt. Letztendlich ergibt sich jeweils für die Normal- und Intensivstation ein Prozentwert der COVID-19-Patienten je Landkreis, für die das Universitätsklinikum zuständig wäre. Auf Basis der aufgenommenen Patienten und der infizierten Patienten im Einzugsgebiet lässt sich die Rate der Patienten abschätzen, die eine Behandlung auf der Normal- oder Intensivstation benötigen. Die Rate für die Normalstation beträgt hierbei aktuell 13,3 % und 3,9 % auf der Intensivstation. Bei den Patientenaufnahmen der COVID-Normalstation stellten die tatsächlich erkrankten Patienten einen Anteil von 31 %, die nichtbestätigten Verdachtsfälle einen Anteil von knapp über 69 %. Auf der Intensivstation lag dieses Verhältnis bei 45 % zu 55 %. Da die negativen Verdachtsfälle für COVID-19 vorgesehene Kapazitäten blockieren, werden diese auch im Modell berücksichtigt, mit jeweils eigenen Verteilungen für die Liegedauer als auch der täglichen Ankunftsrate. Letztere wird aufgrund der Korrelation bei der Aufnahme von neuen Patienten zwischen tatsächlichen COVID-19-Patienten und im Nachhinein negativen Verdachtsfällen auf das 2,1-Fache auf der Normalstation und auf das 1,1-Fache auf der Intensivstation der täglich anzunehmenden COVID-19-Aufnahmen festgelegt. Der letzte benötigte Parameter für das Modell ist die Verweildauer auf der COVID-Normal- bzw. -Intensivstation. Alle Parameter, die in dem Modell verwendet wurden, sind in Tab. 2 abgebildet. Sie basieren u. a. auf realen Daten am Universitätsklinikum Augsburg sowie den historischen Fallzahlen der Neuinfizierten. Die lokalen Daten wurden durch den Pandemiebeauftragten des Universitätsklinikum Augsburg und vonseiten des Katastrophenschutzes durch den Ärztlichen Leiter Führungsgruppe Katastrophenschutz überprüft.

**Table 2:** 

	Min.	Modus	Max.
*Normalstation COVID-19*			
Aufnahmen auf Basis Neuinfizierter	11 %	13 %	15 %
Bleibedauer auf Normalstation (Tage)	1	4	12
*Normalstation Nicht-COVID-19*			
Ankunftsrate auf Basis der COVID-19-Aufnahmen	200 %	210 %	220 %
Bleibedauer auf Normalstation (Tage)	1	2	5
*Intensivstation COVID-19*			
Aufnahmen auf Basis Neuinfizierter	3,6 %	3,8 %	4,0 %
Bleibedauer auf Intensivstation (Tage)	1	5	22
*Intensivstation Nicht-COVID-19*			
Ankunftsrate auf Basis der COVID-19-Aufnahmen	110 %	120 %	130 %
Bleibedauer auf Intensivstation (Tage)	1	3	5

### Prognose der Bettenbelegung

**Figure 1:**
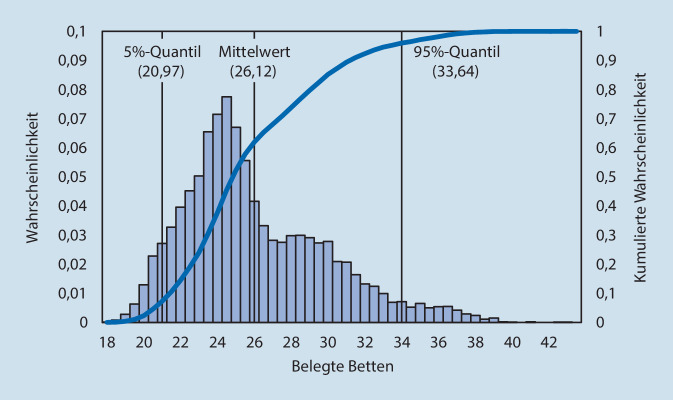

Nach Durchführung der Simulation können die definierten Performance-Kennzahlen genauer betrachtet werden. Eine dieser Kennzahlen ist die Verteilung der Bettenbelegung auf einer Station an einem Tag. Beispielhaft hierfür wird die Wahrscheinlichkeitsverteilung der Prognose für den 12.04.2020 auf der Intensivstation näher erläutert (Abb. 1). Die Simulation selbst wurde am 03.04.2020 durchgeführt.

**Figure 2:**
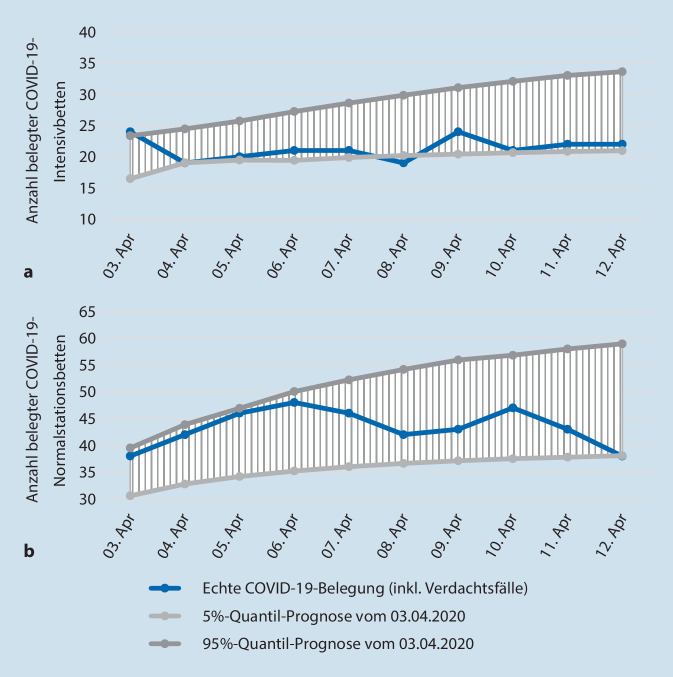

Die Abbildung zeigt die Verteilung der benötigten Betten auf Basis von 10.000 Realisationen. Auf der primären vertikalen Achse, sowie dem Histogramm, kann die Eintrittswahrscheinlichkeit für eine bestimmte Anzahl benötigter Betten abgelesen werden. Auf der sekundären vertikalen Achse wird die Verteilungsfunktion (kumulierte Eintrittswahrscheinlichkeit) abgetragen. Diese wird durch die Linie in der Grafik abgebildet. So werden beispielhaft mit einer Wahrscheinlichkeit von 50 % mindestens 26,12 Intensivbetten für COVID-19-Patienten und Verdachtsfälle benötigt. Des Weiteren sind das 5 %- und 95 %-Quantil sowie der Erwartungswert ersichtlich. Für eine bessere grafische Darstellung über die kurzfristige Entwicklung der Bettenbelegung werden das 5 %- und 95 %-Quantil der Verteilungen von mehreren Tagen im Zeitablauf dargestellt (Abb. 2) und mit der tatsächlich realisierten Belegung auf der Intensivstation im Betrachtungszeitraum verglichen. So wurde in der Prognose vom 03.04.2020 für den 12.04.2020 eine Belegung im Bereich von 20,97 bis zu 33,64 Betten zwischen dem 5 %- und 95 %-Quantil berechnet. Tatsächlich waren am 12.04.2020 22 Betten auf der Intensivstation (Abb. 2a) belegt.

Das gleiche Vorhergehen wurde auch auf der Normalstation durchgeführt. Die Ergebnisse sind in Abb. 2b dargestellt.

Neben den in Abb. 2 gezeigten Prognosen wurden im Laufe der Pandemie weitere Prognosen mit einem 10-tägigen Horizont erstellt. Im Zeitraum vom 28.03.2020 bis zum 08.04.2020 wurden insgesamt 4 Prognosen mit einem 10-Tages-Horizont für die COVID-19-Intensivstation und vom 03.04.2020 bis zum 20.04.2020 zwei Prognosen für die COVID-19-Normalstation am Universitätsklinikum Augsburg erstellt. Die Prognosen haben im Mittel eine Unterschätzung von 0,3 (1,6 % relative Abweichung) Intensivbetten sowie nahezu keine Unterschätzung der Normalstationsbetten (0,1 % relative Abweichung) im Vergleich zur tatsächlich benötigten Kapazität aufzuweisen. Ebenso ist die Überschätzung der benötigten Betten sehr gering und beträgt bei der Intensivstation einer absoluten Abweichung von 0,1 Intensivbetten (0,3 % relativ) und keiner Abweichung bei den Normalstationsbetten. Die Unterschätzung spiegelt die mittlere absolute und relative Differenz zwischen den tatsächlich benötigten Betten sowie dem 95 %-Quantil einer Prognose am jeweils gleichen Tag wider; die Überschätzung die mittlere Differenz zwischen den tatsächlich benötigten Betten und dem 5 %-Quantil einer Prognose. Durch eine enge Zusammenarbeit mit dem Ärztlichen Leiter des Katastrophenschutzes wurde das Simulationsmodell Mitte April 2020 erweitert. Durch die Anpassung ist es möglich, die benötigte Intensiv- und Normalbettenkapazität im Rettungsdienstbereich Augsburg zu prognostizieren. Im Zeitraum vom 08.04.2020 bis 08.06.2020 wurden insgesamt 23 Prognosen für die benötigte Intensiv- als auch Normalbettenkapazität für den Rettungsdienstbereich Augsburg erstellt. Als Input-Parameter dieser Prognosen wurden viele der am Universitätsklinikum Augsburg erhobenen Daten verwendet. Auch hier konnten sehr gute Prognosewerte erzielt werden. Die Erweiterung für den Rettungsdienstbereich Augsburg zeigt ähnlich gute Ergebnisse. Die mittlere Unterschätzung der benötigten Intensivbettenkapazität beträgt 0,23 (5,5 % relative Abweichung) sowie 0,27 (3,4 % relative Abweichung) bei der Normalbettenkapazität. Die mittlere Überschätzung der benötigten Intensivbettenkapazität beträgt 0,09 (2,9 % relative Abweichung) sowie 0,79 Betten (9,4 % relative Abweichung) bei der Normalbettenkapazität.

**Figure 3:**
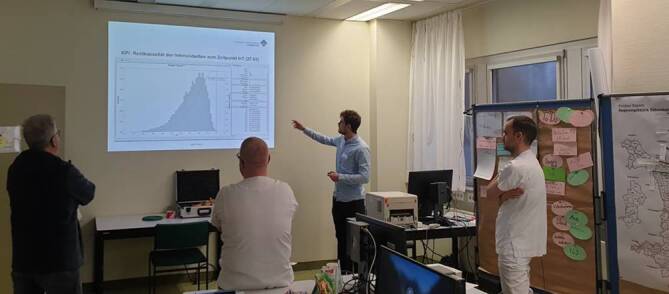

Den realen Einsatz der Methode in der Führungsstelle des Universitätsklinikums Augsburg zeigt Abb. 3.

## Diskussion

### Lokale Daten sind (inter-)national vergleichbar

Unsere retrospektiv erhobenen deskriptiven Daten zeigen die Notwendigkeit einer intensivmedizinischen Betreuung bei 38 % der COVID-19-positiv hospitalisierten Patienten. Eine invasive Beatmung wurde in 19 % der COVID-19-positiv hospitalisierten Patienten bzw. bei 50 % der auf Intensivstation verlegten COVID-19-positiven Patienten notwendig. In der Literatur aus Wuhan zeigt sich die Rate an ARDS in einer Studie an 201 COVID-19-erkrankten hospitalisierten Patienten bei 40 % und damit vergleichbar zu unseren Daten [2]. Die Sterblichkeit der hospitalisierten COVID-19-positiven Patienten lag in unserem Haus kumulativ bei 8 % gesamt bzw. bei 18 % der auf der Intensivstation aufgenommenen Patienten. Diese Zahlen erscheinen soweit bei der geringen Stichprobe mit anderen deutschen Kliniken der Maximalversorgerstufe vergleichbar [3]. Der Anteil von Frauen sowie das Alter der hospitalisierten COVID-19-Patienten scheinen mit 33 % bzw. 65 Jahren ebenso vergleichbar [3]. Hier zeigt sich eine klare Überrepräsentation der Population über 60 Jahren in unserer Klinik, welche in den bisherigen Zahlen des Robert Koch-Instituts einen Anteil von 14 % der Gesamtinfizierten in der Population ausmacht [10]. Jedoch konnte anhand amerikanischer und chinesischer Daten bereits ein Zusammenhang zwischen Hospitalisierungsrate und dem Alter der Patienten gezeigt werden [4, 12].

### Anwendung für die Einsatztaktik

Das hier entwickelte simulationsbasierte Prognosemodell zur kurzfristig benötigten Bettenkapazität ermöglicht, die benötigten Bettenkapazitäten für verschiedene Pandemieverläufe abzuschätzen. Hierdurch können weitere Maßnahmen und die Vorhaltung weiterer Ressourcen frühzeitig eingeleitet werden. Wir konnten für unsere Prognose vom 03.04.2020 zeigen, dass die tatsächliche Anzahl der benötigten Betten für COVID-19-Erkrankte und Verdachtsfälle sowohl auf der COVID-Normalstation als auch auf der COVID-Intensivstation nahezu korrekt vorhergesagt wurde. In beiden Vorhersagen erkennt man, dass die tatsächliche Entwicklung im unteren Bereich der Prognose lag, was vermutlich durch den starken Rückgang der kumulierten Neuinfektionen pro Tag, bedingt durch die von der Regierung erlassenen Kontaktsperren, begründet ist. Dies verdeutlicht die Notwendigkeit einer ständigen Anpassung der Input-Parameter und der wiederholten Durchführung der Prognosen, um die aktuellen Trends der Pandemieausbreitung abzubilden.

### Vergleich zu existierenden Simulationen

Verschiedene Autoren haben sich mit dieser Problematik befasst [6, 7, 9, 13]. Paessler et al. prognostizieren einen Erwartungswert für die zu erwartende Bettenbelegung auf Intensiv- und Normalstationen für eine festgelegte Region auf Basis von deterministischen Parametern [9]. Stang et al. verwenden ein ähnliches deterministisches Prognosemodell, das die benötigte Intensivkapazität in ganz Deutschland für verschiedene Szenarien ohne Eintrittswahrscheinlichkeit berechnet [13]. Meares et al. verwenden ein Warteschlangenmodell, um die benötigten Intensivkapazitäten in Australien zu berechnen. Auch hier werden verschiedene deterministische Szenarien ohne Eintrittswahrscheinlichkeiten vorgestellt [7]. Nach einer ausführlichen Literaturrecherche konnte zum jetzigen Zeitpunkt kein Modell gefunden werden, in der das regionale Einzugsgebiet eines Krankenhauses mit stochastischen Ansätzen zur Abbildung von Unsicherheit kombiniert und somit eine Bettenbelegung auf der Normal- als auch Intensivstation mit jeweiligen Eintrittswahrscheinlichkeiten anhand von Verteilungsfunktionen prognostiziert wird.

Zusätzlich werden bisher in keinem Modell Verdachtsfälle berücksichtigt. Die Betrachtung dieser Patienten ist jedoch wichtig, da diese ebenso wie tatsächliche COVID-19-Patienten auf den vorgesehenen Stationen aufgenommen werden und dadurch die Bettenkapazitäten in Anspruch nehmen.

### Verwendung einfacher Eingabeparameter

Je exakter die Eingabeparameter der Simulation die Realität widerspiegeln, desto besser sind die Prognosen. Aufgrund der rasanten Entwicklung können verfügbare Daten nicht immer den Ansprüchen gerecht werden. Daher wurde das Modell für möglichst wenige Eingabeparameter konzipiert, die zudem vergleichbar leicht zu beschaffen sind, um robuste Resultate zu erhalten. Ausgenommen ist dabei die Entwicklung an kumulierten Neuinfektionen pro Tag, die den größten Einfluss auf die Simulationsergebnisse hat. Der Zeitpunkt, ab dem weitere (politische) Maßnahmen, wie z. B. die verhängte Kontaktsperre, greifen und sich auf die Bettennachfrage in Krankenhäuser tatsächlich auswirken, ist schwer vorherzusagen. Durch fortlaufende Anpassungen der Datengrundlage und neu durchgeführte Simulationen können die Vorhersagen aber weiter geschärft werden.

### Ausblick

Nach erfolgreicher Implementierung am Universitätsklinikum Augsburg sowie dem Rettungsdienstbereich Augsburg ist eine landesweite Ausweitung geplant. Diese kann in Absprache mit den Verantwortlichen des bayerischen Gesundheitsministerium erfolgen.

#### Kernaussagen

- Für die Krankenhäuser liegt eine der größten Herausforderungen bei der COVID-19-Pandemie in der Abschätzung der benötigten Bettenkapazitäten.
- Als Hilfestellung zur Abschätzung von benötigten Bettenkapazitäten für COVID-19-Patienten sowie Verdachtsfälle wurde ein simulationsbasiertes Prognosemodell entwickelt.
- Die notwendigen Parameter sind die Wachstumsrate an kumulierten Neuinfektionen pro Tag, der Anteil der hospitalisierten Neuinfizierten, die Verweildauer sowie das Einzugsgebiet.
- Unser simulationsbasiertes Prognosemodell zur kurzfristigen Kapazitätsbestimmung ermöglicht eine valide Abschätzung der benötigten Bettenkapazitäten für verschiedene Pandemieverläufe.
- Sollten die politischen Vorgaben bezüglich Kontakt- und Ausgangssperren geändert werden, könnte der zukünftige Verlauf der benötigten Bettenkapazitäten exakter prognostiziert werden, aufgrund der sich dann im historisch erfassten Korridor bewegenden Wachstumsrate an kumulierten Neuinfektionen.
